# Same-day discharge after percutaneous coronary procedures—Structured review and comprehensive meta-analysis

**DOI:** 10.1007/s00508-024-02347-z

**Published:** 2024-05-14

**Authors:** Mathias C. Brandt, Hannes Alber, Rudolf Berger, Ronald K. Binder, Julia Mascherbauer, Alexander Niessner, Martin Schmid, Bernhard Wernly, Matthias Frick, Austrian working group of interventional cardiology

**Affiliations:** 1 Department of Internal Medicine II, Paracelsus Medical University, Salzburg, Austria; 2 Department of Cardiology, Public Hospital Klagenfurt am Woerthersee, Klagenfurt am Woerthersee, Austria; 3 Department of Internal Medicine, Brothers of Saint John of God Eisenstadt, Eisenstadt, Austria; 4 Department of Cardiology and Intensive Care, Klinikum Wels, Wels, Austria; 5 Department of Internal Medicine 3/Cardiology, University Hospital St. Pölten, Karl Landsteiner University of Health Sciences, Krems, Austria; 6 Department of Internal Medicine II, Division of Cardiology, Medical University of Vienna, Waehringer Guertel 18–20, 1090 Vienna, Austria; 7 Department of Cardiology, Ordensklinikum Linz Elisabethinen, Linz, Austria; 8 Department of Internal Medicine, General Hospital Oberndorf, Teaching Hospital of the Paracelsus Medical University, Salzburg, Austria; 9 Department of Internal Medicine I and Cardiology, Teaching Hospital Feldkirch, Feldkirch, Austria

**Keywords:** Outpatient treatment, Interventional cardiology, Ambulatory coronary intervention, Structured review, Meta-analysis

## Abstract

**Introduction:**

Percutaneous coronary intervention is a well-established revascularization strategy for patients with coronary artery disease. The safety and feasibility of performing these procedures on a same-day discharge basis for selected patients has been studied in a large number of mostly nonrandomized trials. An up to date literature review should focus on trials with radial access, representing the current standard for coronary procedures in Austria and other European countries.

**Methods:**

The aim of this consensus statement is to review the most recent evidence for the safety and feasibility of performing same-day discharge procedures in selected patients. A structured literature search was performed using prespecified search criteria, focusing on trials with radial access procedures.

**Results:**

A total of 44 clinical trials and 4 large meta-analyses were retrieved, spanning 21 years of clinical evidence from 2001 to 2022. The outcome data from a wide range of clinical settings were unanimous in showing no negative effect on early (24 h) or late (30 day) major adverse events after same-day discharge coronary procedures. Based on nine prospective trials a comprehensive meta-analysis was compiled. Using 1‑month major adverse events data the pooled odds ratio of same-day discharge versus overnight stay procedures was 0.66 (95% confidence interval, CI 0.35–01.24; *p* = 0.19; I^2^ 0%), indicating a noninferiority in carefully selected patients.

**Conclusion:**

Outcome data from same-day discharge coronary intervention trials with radial access confirm the robust safety profile showing no increase in the risk of major adverse events compared to overnight stay.

## Introduction

Recent technical advances such as radial access, third generation drug-eluting stents and highly effective antiplatelet therapy have substantially improved the safety profile of percutaneous coronary procedures (PCP), despite a steady shift towards older, generally sicker patients and more complex procedures [1, 2]. Consequently, catheterization laboratories around the world have implemented outpatient clinics with patient discharge on the same day of the procedure (SDD). As detailed in the position paper of the Austrian working group of interventional cardiology [3] based on the present review, large PCI registries from the USA [4], Canada [5] and France [6] have shown a steady increase in SDD PCI procedures from 10–15% before 2010 to frequencies of 30–45% in 2015–2017. This trend runs parallel with an increase in radial access PCI [1], which has shown a substantially lower rate of bleeding complications and adverse events [7] and is clearly preferred by patients [8]. Currently available data from predominantly nonrandomized or single-center studies show no adverse effects of SDD PCP on short-term or long-term outcome [9–11]. The consistency with which a multitude of studies published from the late 1980s until today, spanning a giant leap from femoral access, bare-metal stent practice to radial access and drug-eluting stents, was able to confirm no added risk for selected patients with stable coronary artery disease (CAD) is very impressive. Nevertheless, there are still concerns about SDD procedures [9]. The most important doubt is the question if major complications can be detected with adequate precision, even including large registries, due to their very low event rate. As pointed out before [9, 10], most single-center studies are underpowered to detect rare adverse events, therefore meta-analyses are of specific importance as a foundation for practice recommendations. Currently available meta-analyses have been published over a large time span [9–12], therefore incorporating a relatively heterogeneous group of technical, interventional and medication standards. Especially the fact that currently radial access is the clinical standard for diagnostic angiographies (DA) and coronary interventions (PCI) in Austria and many other European countries would make a review and meta-analysis with a focus on data from radial access SDD procedures especially valuable.

While the majority of centers in Austria still currently schedule at least one mandatory overnight stay after the procedure as the clinical standard of care, procedures with discharge on the same day are performed with increasing frequency. With respect to constantly rising numbers of coronary interventional procedures, staff shortages on regular wards and pressure for cost reduction, an expansion of ambulatory interventional cardiology programs appears to be an intriguing option. It should be noted that those clinics in Austria running SDD programs confirm a favorable safety profile for outpatient PCP and a high level of patient satisfaction when allowed to leave the outpatient clinic 3–6h postprocedure.

The goal of this meta-analysis is to review the most recent evidence regarding the safety and feasibility of performing SDD serving as a foundation for the corresponding position paper by the Austrian Society of Cardiology on this subject [3].

## Methods

### Literature review

**Fig. 1:**
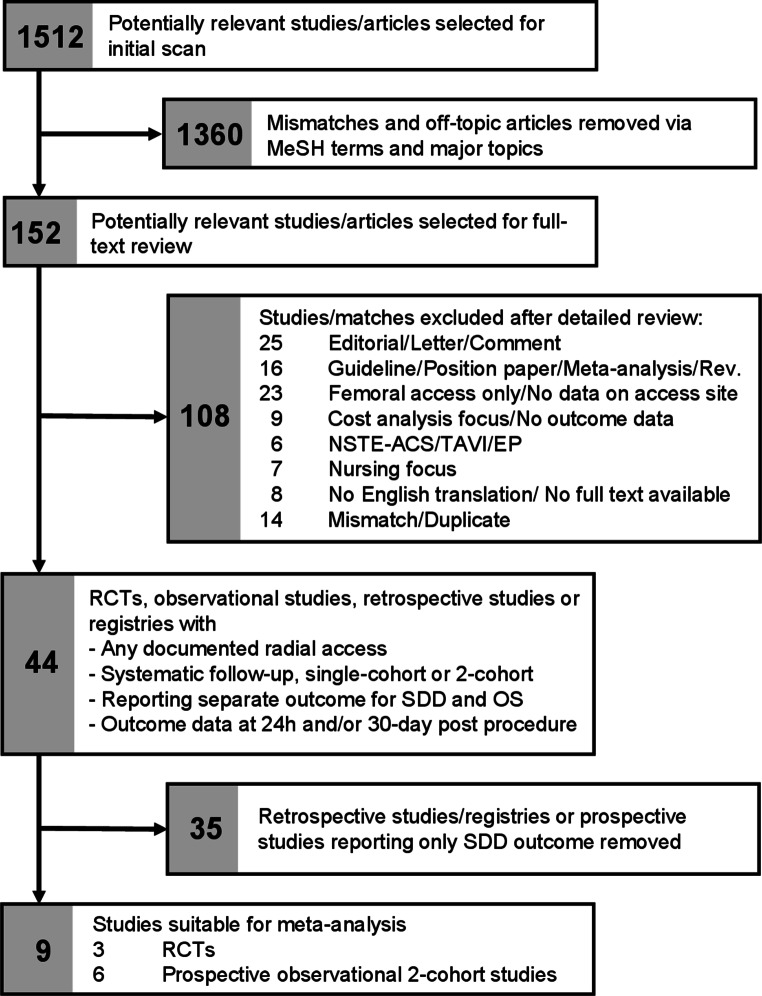
PRISMA flow diagram showing literature search strategy and filtering process for use in meta-analysis. *RCT* randomized controlled trial, *NSTE-ACS* non-ST-elevated acute coronary syndrome, *TAVI* transaortic valve implantation, *EP* electrophysiology. (adapted from [9])

We conducted a literature search on EMBASE, PubMed and CINAHL following the general recommendations from the Preferred Reporting Items for Systematic Review and Meta-Analysis (PRISMA) [13] for randomized trials, and the Cochrane Collaboration and Meta-Analyses of Observational Studies in Epidemiology (MOOSE) group for observational studies [14]. For the search we used the MeSH search terms (“same-day discharge” OR “outpatient” OR “ambulatory” OR “daycare”) AND (“coronary artery” OR “PCI” OR “intervention” OR “angiography”) NOT (“psychiatric” OR “orthopedic” OR “vascular” OR “peripheral”). As detailed in Fig. 1, from the initial 1512 potentially relevant titles 1360 mismatches were removed by further filtering. From the remaining 152 references, 23 studies with exclusively femoral access or without exact numbers on access sites for both SDD and OS patients were excluded. Further 25 editorials/comments or letters and 16 studies with a cost-analysis or nursing focus were removed leaving 44 studies with either a prospective (Table 1) or retrospective design (Table 2), with specific data on radial access in SDD PCI and reporting outcome data for 30-day and/or 24‑h follow-up. For the meta-analysis, 9 prospective trials (three RCTs and six 2-cohort studies) reporting separate outcomes for SDD and OS were extracted.

**Table 1:** Prospective clinical trials on SDD PCI with radial access

Publication (Reference)	Study type	Sample size (*n*)	Radial access (%)	Clinical setting*	Country	Excluded or switch to OS (%)	Major complications 6–24 h	Primary endpoint	Outcome
1. Slagboom et al., 2001 [53]	Observational, prospective, single arm	Total: 159	SDD: 100%	Elective PCI, stable and unstable AP. Lesion type C (13%), SVG (3%)	Netherlands	35%	None	Death, MI, stroke, TVR, bleeding complications, rehospitalization	No rehospitalizations
		SDD: 106							No adverse events 6–24 h
2. Kumar et al., 2004 [19]	Observational, prospective single arm	120	Total: 97.3%	Elective PCI, MV (19.4%), lesion type C (27.5%), multistent, SVG (1.7%)	UK	20%	0.8% (1x stent thrombosis)	MACE (death, MI, stroke, TVR, rehospitalization) at 30 days	MACE at 30 days 0.67%
			SDD: 100%						
3. Oh et al., 2004 [47]	Observational, prospective, single arm	Total: 230	SDD: 100%	Elective PCI, MV (8.3%), lesion type C (24.3%)	South Korea	10%	None (1% minor hematoma, bleeding)	MACE (death, MI, stroke, TVR, CABG, major bleeding)	0–24h: 1% hematoma, 1% bleeding. 0% MACE. After 7 days: 0.5% hematoma, 0% MACE
		SDD: 206							
4. Slagboom et al., 2005 [18]	Observational, prospective, randomized radial vs. femoral access	Total: 644	SDD: 52.8%	Elective PCI, multivessel PCI, lesion type C (18%), SVG (2%)	Netherlands	42%	0.26% (1x stent thrombosis)	Primary: death, MI, emergency CABG, re-PTCA, readmission, entry site complication, major bleeding at 24 h. Secondary: patient comfort, cost-effectiveness	SDD: 0.26% MACE (1 stent thrombosis)
		SDD: 375	OS: 46.0%						OS: 8.17% MACE (1 death, 13 MI, 2 re-PTCA, 7 emergency CABG)
5. Bertrand et al., 2008 [54]	RCT, prospective	SDD: 504	SDD: 100%	Elective and acute PCI, complex PCI, NSTEMI	Canada	12%	None	Death, MI, revascularization, major bleeding, thrombocytopenia, access site complication, rehospitalization at 30 days	No difference composite 30-day (1.4% vs. 1.8%), 6‑month, 1‑year EP
		OS: 501	OS: 100%						SDD had abciximab bolus only
6. Chaumeil et al., 2008 [55]	Observational, prospective, single arm	130	SDD: 97.1%	DA, Elective PCI, ad hoc PCI, bifurcation (26.1%), LM (1.4%)	France	24.4%	None (3% minor complic.)	Death, MI, stroke, repeat PCI, emergency bypass surgery, major bleeding, allergic reaction	No rehospitalizations
									No adverse events 6–24 h
									1x allergic reaction, 1x forearm edema, 1x chest pain
7. Jabara et al., 2008 [23]	Observational, prospective, single center	SDD: 12 OS: 438	SDD: 100%	Elective and acute PCI, NSTEMI, STEMI (3%), MV (31%), lesion type C (8%), bifurcation (7%), CTO (1.4%), LIMA/SVG (3%)	USA	SDD only selected patients	None	Death, MI, TLR, access site complications	OS: adverse events in 5.4%, 4.4% 0–6h; 0.9%> 24h
			OS: 100%						No adverse events 6–24 h
8. Chung et al., 2010 [34]	Observational, prospective, parallel group	SDD: 214 OS: 446	SDD: 100%	Elective PCI	Taiwan	ND	None	Death, MI, stroke, emergency CABG, post-PCI angina, vascular access complications	No difference in 30-day MACE SDD vs. OS: 0.2% vs. 1.4%
			OS: 100%	Ad hoc PCI, MV (2.8%), RA (0.9%)					
9. Herman, 2011 [56]	Observational, prospective, single arm	130	SDD: 82%	Elective PCI, MV (21%), CTO (9%)	New Zealand	0%	None	Death, MI, TVR, re-hospitalization	No deaths within 30 days, No rehospitalizations
									No adverse events 6–24 h
10. Le Corvoisier et al., 2013 [49]	Observational, prospective, single arm	220	SDD: 100%	Elective PCI, MV (14.1%), bifurcation (21.5%), multi-stent, CTO (8.6%)	France	3.2%	None	Death, MI, TVR, readmission, bleeding, patient anxiety, patient satisfaction at 24 h and 30 days	No MACCE within 24 h. 1 Patient with MI at 30 days
									87% patient satisfaction
11. Hodkinson et al., 2013 [26]	Observational, prospective, single arm	1059	SDD: 98.1%	Elective and acute PCI, complex, MV (12.7%), bifurcation (31.4%), LM (3.5%)	Ireland	ND	None	MACE (death, stroke, MI, TVR), vascular complications at 30 days	MACE 0.89% at 30 days
12. Muthusamy et al., 2013 [57]	Observational, prospective, single arm	200	SDD: 25%	Elective PCI, MV (7.5%), protected LM (0.5%)	USA	ND	None (4% minor bleeding)	MACE (death, stroke, MI, TLR, TVR, major bleeding, vascular complications)	MACE: 0–24h 0%; 1–7 days 0%
									75% femoral access
13. Aydin et al., 2014 [58]	Observational, prospective, single arm	254	SDD: 100%	Elective PCI, complex, lesion type C (45.2%), LIMA/SVG (4.7%), CTO (10.2%)	Turkey	39%	None	Death, MI, major bleeding, local hematoma, aneurysm, arteriovenous fistula	MACE: 4 MI (1.6%) 0–2h; 4 MI (1.6%) > 24h. Minor bleeding: 8 (3.1%) 0–2h
									Timing of complications: 54.2% 0–2h, 0% 2–24h, 45.8% > 24h
14. Saad et al., 2015 [50]	Observational, prospective, 2‑cohort	SDD: 149 OS: 154	SDD: 28%	Elective PCI, lesion type C (30.2%), MV (45.6%), LM (0.7%)	Australia	90% (predefined SDD criteria)	None	Death, MI, stroke, TVR, major bleeding, unplanned rehospitalization. Significance of post-PCI trop‑T levels	30-day: rehospitalization 3.4% (SDD) vs. 0.7% (OS; *p* = n. s.)
			OS: 4%						16 months composite MACE: 6.1% (SDD) vs. 6.0% (OS; *p* = n. s.)
15. Singh et al., 2015 [59]	Observational, prospective, matched pair	SDD: 56	SDD: 96.6.%	Elective PCI, complex, MV (44.6%), CTO (3.6%)	India	8.2%	None	MACCE (cardiac death, stroke, MI, repeat PCI, urgent bypass surgery)	SDD: 1 patient stent thrombosis, 1 patient chest pain within 6 h, OS: 1 patient stent thrombosis within 6 h
		OS: 56	OS: 100%						
16. Cordoba-Soriano et al., 2017 [60]	Observational, prospective, multicenter	Total: 723	SDD: 99%	Elective PCI, MV (24.6%)	Spain	26%	None	MACE (death, MI, stent thrombosis, TLR, TVR, stroke, major bleeding, AKI, vascular complications, access site complications) at 24 h and 30 days	24 h MACE: 0.19% (1 rectal bleeding unrelated to access site); 30-day MACE: 0.56% (1 stent thrombosis, 1 re-PCI, 1 stroke)
		SDD: 533	OS: 93%						
		OS: 190							
17. Amin et al., 2018 [35]	Observational, prospective, single center	SDD: 230	SDD: 42%	Elective PCI, bifurcation (18%), MV, CTO (8%), RA (2%)	USA	ND	ND	MACE (death, stroke, MI, AKI, TVR, major bleeding, vascular complications)	MACE at 30 days: 0.43% (SDD) vs. 11.56% (OS)
		OS: 1522	OS: 5%						
18. Rodriguez-Araujo et al., 2018 [61]	Observational, prospective, 2‑cohorts	SDD: 245	SDD: 100%	Elective PCI, low risk	USA	0%	ND	MACE (death, stroke, MI, TVR, renal failure) or cardiovascular complications, financial costs at 30 days	No difference In MACE SDD vs. OS: All-cause mortality (0% vs 0%), MI (0% vs. 0.08%), reintervention (2.5% vs. 2.1%), procedural complications (3.7% vs. 2.5%)
		OS: 245	OS: 100%						
19. Cordoba-Soriano et al., 2019 [28]	Prospective registry, multicenter Complex vs. simple PCI	SDD: 791	SDD: 99.8%	Elective PCI, complex, MV (16.2%), bifurcation (1.4%), CTO (3.3%), RA (1.1%), LM (1.0%)	Spain	24.5%	None (< 1% hematomas)	MACE (death, MI, TLR, TVR, non-TVR, major bleeding, stroke, AKI, major vascular complications) at 24 h and 30 days	Complex PCI: 0% MACE at 24 h and 30 days. Simple PCI: 0.17% at 24 h, 0.68% at 30 days
		OS: 256	OS: 94.6%						
20. Rodrigues et al., 2020 [22]	Observational, prospective, single center	SDD: 43	SDD: 91%	Elective PCI, MV (4.7%), bifurcation (16.3%), protected LM (2.3%)	Portugal	0.65%	None	Primary EP: MACE (death, stroke/TIA, urgent revascularization, vascular complication) at 4–24h and 1–30 days	30-day MACE: SDD 0% vs. OS 0% (*p* = n. s.)
		OS: 111	OS: 59%					Secondary EP: unplanned hospital visit, readmission, recatheterization	Only minor adverse events detected
21. Gaba et al., 2021 [21]	RCT, prospective	SDD: 100	SDD: 20%	Elective LM-PCI, MV	USA	1.3%	ND	30-day MACE (death, MI, Stroke, TVR, stent-thrombosis)	30-day MACE: SDD 4%, OS 5% (*p* = 0.38)
		OS: 835	OS: 27%						
22. Kaur et al., 2022 [36]	Observational, prospective, single center	Total: 675	SDD: 82%	Elective PCI, ACS, lesion type C (63%), bifurcation (15.2%), CTO (5.3%), RA (2.3%), LM (0.2%)	18%	India	None	MACE at 30 days: death, stroke, MI, TVR, bleeding, rehospitalization	30 days to 6 weeks: SDD 0% vs. OS 0.61% (*p* = n. s.)
		SDD: 132	OS: 52%						
		OS: 485							

Overview of prospective clinical trials on SDD PCP outcome with focus on radial/ulnar access

*ACS* acute coronary syndrome, *AKI* acute kidney injury, *DA* diagnostic angiography, *EP* endpoint, *CABG* coronary artery bypass graft, *CI* confidence interval, *CIN* contrast-induced nephropathy, *CTO* chronic total occlusion, *LIMA* left internal mammary artery, *MACE* major adverse cardiovascular events, *MACCE* major adverse cardiovascular or cerebral events, *MI* myocardial infarction, *MV* multivessel PCI, *SDD* same-day discharge, *SVG* saphenous vein graft PCI, *OS* overnight stay, *RA* rotational atherectomy, *RCT* randomized controlled trial, *OBS* observational studies, *PCI* percutaneous coronary intervention, *LM PCI* left main coronary artery PCI, *PTCA* percutaneous transcoronary angioplasty, *TIA* transient ischemic attack, *TLR* target lesion revascularization, *TVR* target vessel revascularization, *n.* *s.* no significant differences, *ND* no data available

* Percentages refer to the SDD cohort if not specified otherwise

**Table 2:** Retrospective clinical trials on SDD PCI with radial access

Publication (reference)	Study type	Sample size (*n*)	Radial access (%)	Clinical setting*	Country	Excluded or switch to OS (%)	Major complications 6–24 h	Primary endpoint	Outcome
1. Ziakas et al., 2003 [62]	Observational, retrospective, single arm	943	SDD: 100%	Elective PCI, lesion type C (17%), MV (16%)	Canada	14%	None (2.8% minor)	Death, MI, TVR, rehospitalization, access-site complications at 24 h and 30 days	0–24h: 2.8% minor access site complications, 2% chest pain
									30 days: 1.3% repeat angio, 0.4% stent thrombosis
2. Wiper et al., 2006 [63]	Observational, retrospective, single arm	SDD: 377	SDD: 94%	Elective PCI, MV (33%), lesion type C 28%, bypass graft (3%)	UK	15%	None	Death, Angina, TLR, heart failure, major/minor bleeding, intracranial bleeding, hematuria at 30 days	SDD: 1 Cardiac death 72 h post procedure, 2 STEMI
		OS: 65	OS: 61%						48 h post procedure (stent thrombosis), 9% hematoma
									OS strategy only if peri-procedural adverse events
									30-day MACE 0.68%
3. Small et al., 2007 [27]	Observational, retrospective	SDD: 1174	SDD: 100%	PCI: Emergent 29%, urgent 54%, MV (23%), LM (2%)	USA	ND	None	Safety: death, stroke, MI, revascularization, major/minor bleeding	OS had higher risk patients with more frequent complications necessitating observation. 0.6% Urgent bypass, 0.6% PE, 0.5% bleeding, 1.2% death
	Higher risk vs. lower risk patients	OS: 1015	OS: 100%						No adverse events 6–24h
4. Perret et al., 2009 [37]	Observational, retrospective, single center	Total: 3136	SDD: 80%	Elective PCI, complex (49%), bifurcation (13%), CTO (12%), RA (2%)	France	5.9%	None	MACE (death, MI, stroke, emergency re-PTCA, major bleeding) 0–6h, 6–24h, 1–3 days, 3–30 days postprocedure	30-day MACE: SDD 3.9%, no events within 6–24 h postprocedure. No comparator from OS patients for MACE
		SDD: 95	Total: 17%						
5. Gilchrist et al., 2012 [64]	Observational, retrospective, single arm	Total: 665	Total: 84%	Elective PCI, complex (22%), MV (3%), LM (5%), SVG (2%)	USA	0%	None	Death, MI, TVR, acute/late re-hospitalization	No deaths within 30 days, no rehospitalizations
		SDD: 100	SDD: 100%						No adverse events 6–24 h
6. Koutouzis et al., 2017 [25]	Observational, retrospective	SDD: 28	SDD: 82.1% (ulnar 17.8%)	Elective PCI, single vessel, multistent	USA	ND	None	MACE (death, stroke, MI, TVR), major bleeding, stent thrombosis at 30 days	MACE 0% in 28 Pat. SDD
		OS: 138	OS: 50%		Greece				MACE complex vs. noncomplex 3% vs. 0.7%
7. Amin et al., 2018 [15]	Retrospective, multicenter, cohort study	Total: 672,470	SDD: 8.9%	Elective PCI, complex, MV, lesion type C (69%), bifurcation (18%), CTO (8%), RA (2%)	USA	ND	ND	Death, MI, AKI, major bleeding	30-day: death OR SDD 0.29 (95% CI 0.14–0.63) vs. OS 1.82 (95% CI 1.68–1.98)
		SDD: 60,920	OS: 3.4%						90-day: death OR SDD 1.60 (95% CI 1.20–2.12) vs. OS 3.99 (95% CI 3.74–4.26)
8. Rubimbura et al., 2018 [6]	Observational, retrospective	SDD: 1073	SDD: 98.5%	Elective PCI, MV (11.2%), bypass graft (1.2%)	France	34%	None	Prim. EP: MACCE (death, MI, stroke, repeat PCI, urgent cardiac surgery, major vasc. complication)	3.7% adverse events during PCI, 3.2% adverse events 0–6h post-PCI > OS, SDD: No MACCE within 24 h
		OS: 562	OS: 89%					Sec. EP: readmission within 24 h	
9. Madan et al., 2019 [5]	Observational, retrospective, longitudinal	SDD: 10,801	SDD: 56.4%	Elective PCI (41.5%), ad hoc PCI (58.0%), MV (24.4%)	Canada	ND	ND	Primary EP: All-cause mortality, MI, ACS, rehospitalization at 30 days and 1 year	30-day primary EP: SDD 1.3% vs. 1.6% OS (HR 0.84; 95% CI 0.65–1.08). 30-day mortality SDD 0.1% vs. OS 0.2% (HR 0.40; 95% CI 0.19–0.84)
		OS: 25,171	OS: 37.5%						1‑year primary EP: SDD 6.5% vs. OS 7.6% (HR 0.85; 95% CI)
10. Rymer et al., 2019 [65]	Retrospective, multicenter	Total: 21,261	SDD: 53.2%	Elective PCI	USA	42.3%	ND	30-day mortality, readmission, 30-day accumulated mean costs	Propensity score matching. 30-day mortality: 0% SDD vs. 0.07% OS (*p* = 0.99). 30-day readmission 6.7% SDD vs. 5.6% OS (*p* = 0.24). Cost reduction median 1503 $ (CI 738–2250 $)
		SDD: 728	OS: 20%						
		OS: 1456							
11. Amin et al., 2020 [66]	Observational, retrospective	SDD: 539	SDD: 72.4%	Elective PCI, bifurcation, multistent, CTO (13.9%), protected LM (8.3%)	USA	22%	ND	Feasibility EP: success of SDD	Feasibility EP: SDD in 78%
		OS: 152	OS: 59.2%					Safety EP: MACCE (death, stroke, MI, revasc), bleeding, AKI at 30 days	Safety EP: no difference SDD vs OS (3.2% vs. 3.5%; *p* = 0.195)
12. Ghanbari et al., 2020 [67]	Observational, retrospective, single center	Total: 876	SDD: 29%	NSTE-ACS, lesion type A, B1, B2, C	Denmark	ND	None	MACE: death, stroke, MI, revascularization, vascular complications, CABG, rehospitalization, bleeding	30-day MACE: SDD 1.5% vs. OS 1.4%
		SDD: 190	OS: 21%						OS: 91% of early adverse events within 9 h postprocedure
		OS: 686							
13. Gokhale et al., 2020 [68]	Retrospective, single center	Total: 496	SDD: 100%	Elective PCI, single vessel, bifurcation, RA	USA	ND	None	Primary: MACE (death, MI, stroke), TVR at 30 days	30-day MACE: SDD 0% vs. OS 1.3% (*p* = 0.5)
		SDD: 76	OS: 100%					Secondary: unscheduled medical contact	
		OS: 138							
14. Liew et al., 2020 [20]	Observational, prospective, multicenter	SDD: 586	SDD: 67.1%	Elective PCI, complex (47.6%), MV (5.6%), LM (0.5%)	Australia	ND	ND	30-day MACCE (death, MI, TLR, major bleeding), average costs	30-day MACCE: 0.7% SDD vs. 1.7% OS (*p* = 0.253), 30-day rehospitalization: 0.7% SDD vs. 1.8% OS (*p* = 0.159)
		OS: 17,515	OS: 45.5%						Favorable patient perception
15. Taxiarchi et al., 2020 [24]	Longitudinal, retrospective, multicenter	SDD: 2019	SDD: 24.1 to 58.3%	Elective LM PCI	UK	ND	ND	Mortality at 30 days	SDD: OR 0.70; 95% CI 0.30–1.65 in overall LM PCI, OR 0.48; 95% CI 0.17–1.41 in unprotected LM
		OS: 4433	OS: 17.8 to 51.0%						OS: OR 0.58; 95% CI 0.25–1.34
16. Bradley et al., 2021 [4]	Observational, retrospective, multicenter	SDD: 114,461	SDD: 48.6%	Elective PCI, bifurcation, CTO, LM (1.9%)	USA	15.7%	ND	30-day mortality	30-day mortality: SDD 0.2% vs OS 0.2% (*p* = n. s.)
		OS: 704,630	OS: 19.0%						
17. Chan et al., 2021 [69]	Retrospective, single center	SDD: 106	SDD: 97%	Elective PCI, MV, multistent	Hong-Kong	ND	None	30-day MACE (death, MI, stent thrombosis, hypotension, AKI, acute liver failure, major bleeding, hematoma) at 24 h and 30 days	24 h MACE: SDD 0% vs. OS 10.8%. 30-day MACE: no difference between groups, no exact numbers presented
		OS: 574	OS: 80%						
18. Koutouzis et al., 2021 [40]	Retrospective, single center	Total: 173	SDD: 100%	Elective CTO PCI	Greece	70%	None	30-day MACE (death, MI, stroke, TVR, urgent bypass graft surgery, cardiac tamponade requiring pericardiocentesis or surgery, major bleeding, CIN)	In-hospital MACE: SDD 0% vs. OS 1.6% (*p* = 1.00)
		SDD: 51	OS: 83%						30-day MACE: SDD 0% vs. OS 1.6% (*p* = 1.00)
		OS: 122							
19. Taxiarchi et al., 2021 [38]	Retrospective, multicenter, longitudinal	SDD: 1201	SD: 48%	Elective RA PCI, MV (14%), CTO (10%), LM (8.0%)	UK	6%	ND	30-day MACE	30-day MACE: SDD 0.5% vs. OS 0.35% (*p* = 0.409)
		OS: 3390	OS: 30%						
20. Abdel-Razek et al., 2022 [42]	Observational, retrospective	SDD: 267	SDD: 46.8%	Elective LM PCI	Canada	ND	None (3.4% > 48h)	Death, MI, rehospitalization at 30 days	Prim. composite EP significantly lower in SDD (OR 4.3; 95% CI 1.1–6.0)
		OS: 194	OS: 34.5%						No adverse events < 48h
21. Hariri et al., 2022 [16]	Observational, retrospective, single center	Total: 2529	SDD: 92%	NSTE-ACS, complex, MV, LM (4%)	USA	45% (1144 excluded)	ND	Primary: unplanned all-cause hospital readmission at 30 days, bleeding, mortality at 30 days and 1 year	30-day: readmission 7% SDD vs. 11% OS (*p* = 0.06). Bleeding 0% SDD vs. 2% OS (*p* = 0.02). Mortality 0.3% SDD vs. 0.2% OS (*p* = 1.0)
		SDD: 300	OS: 84%						
		OS: 1085							
22. Taxiarchi et al., 2022 [39]	Observational, retrospective, multicenter	Total: 21,330	SDD: 47%	Elective CTO PCI, complex	UK	ND	ND	30-day mortality	30-day mortality: 0.12% SDD vs. 0.31% OS (*p* = 0.01)
		SDD: 7567	OS: 28%						
		OS: 13,763							

Retrospective clinical trials on SDD PCP outcome with focus on radial/ulnar access. *ACS* acute coronary syndrome, *AKI* Acute kidney injury, *CI* Confidence interval, *CIN* Contrast induced nephropathy, *CTO* Chronic total occlusion, *EP* Endpoint, *LM PCI* Left main PCI, *MACCE* Major adverse cardiovascular or cerebral events, *MACE* Major adverse cardiovascular events, *MI* Myocardial infarction, *MV* Multivessel PCI, *n.* *s.* No significant differences, *ND* No data available, *OBS* Observational studies, *OS* Overnight stay, *PCI* Percutaneous coronary intervention, *RA* Rotational atherectomy, *RCT* Randomized controlled trial, *SDD* Same-day discharge, *TLR* Target lesion revascularization, *TVR* Target vessel revascularization, *UK* United Kingdom, *USA* United States of America

* Percentages refer to the SDD cohort if not specified otherwise

### Comprehensive meta-analysis

**Fig. 2:**
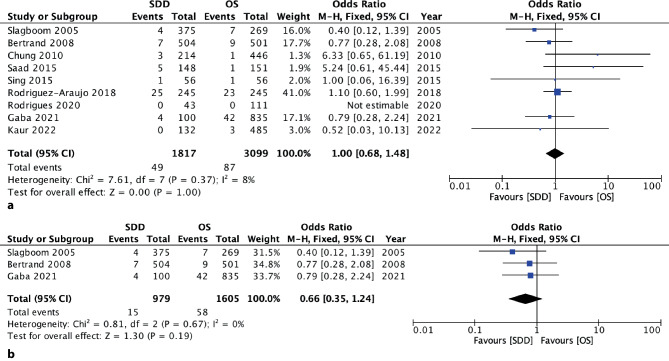
Results of a systematic meta-analysis of prospective trials on the outcome of SDD strategy after PCI. Data show the incidence of MACE at 30 days postprocedure using a Mantel-Haenszel random effects and fixed effects model. (adapted from [9]). **a** Including all prospective trials (3 randomized and 6 nonrandomized) with a total of 1817 SDD cases, **b** including only randomized controlled trials with a total population of 979 SDD patients

This meta-analysis was performed following a standardized protocol by collecting information on study design, sample size, population demographics, coronary angiographic characteristics, access site for PCI, procedural adjuvant pharmacotherapy, procedural success rate, outcome (MACE), and follow-up data. Studies reporting only a single cohort, retrospective studies, and sources not reporting dedicated outcome for 30 days with absolute numbers were excluded from the analysis. Study level data were analyzed. The dichotomous MACE data for the SDD and OS groups were entered into a statistical software program (the Cochrane Collaboration’s Review Manager [RevMan], version 5.4.1, Nordic Cochrane Centre, Copenhagen, Denmark). Heterogeneity across studies was assessed using the Cochran’s Q statistic and the I^2^ statistical test, with values of 25% or less considered low heterogeneity, 25–50% considered moderate, and values over 50% considered substantial. We calculated pooled ORs using a fixed effect model with the Mantel-Haenszel method in cases of low statistical heterogeneity and a random effect model in cases of moderate and substantial heterogeneity (see Fig. 2). The dichotomous outcomes were reported as odds ratios (OR) with their 95% confidence intervals (CI). The weight of each trial on the overall results was calculated as a percentage of the total number of patients included in each outcome analysis. Additionally, a sensitivity analysis was performed based on study design (randomized versus randomized and observational studies).

## Results

### Review of clinical trials

As shown in Fig. 1 the literature search provided 44 clinical trials on SDD PCI with a focus on radial access. Outcome data were analyzed either within a single SDD cohort or in comparison with OS patients. The 22 prospective trials and RCTs containing a total of 5804 patients treated as SDD are shown in Table 1, 22 retrospective trials with 206,517 SDD patients in Table 2. Of the SDD procedures in the 22 prospective trials listed 91% were performed via radial access, in the group of 22 retrospective trials the percentage was lower (39%), mostly caused by 2 large registries with low radial access rates [4, 15]. Calculating from the remaining 20 trials the percentage was 60%. The 44 trials listed are consistent in demonstrating a favorable safety profile of radial access SDD procedures, despite the fact that some studies were performed with routine administration of glycoprotein GPIIb/IIIa antagonists and comprised a wide range of interventional techniques including complex PCIs like multivessel (MV), multistent, bifurcation, CTO or left main coronary artery (LM) interventions. The largest group of interventions in the majority of studies were elective and ad hoc PCIs. In prospective trials, 30-day MACE ranged from 0 to 3.2%, whereas the majority of trials reported MACE rates of 0 or < 1% (see Table 1). In retrospective trials MACE ranged from 0 to 1.5% (see Table 2). In one trial reporting SDD outcomes after NSTE-ACS, 30-day readmission was substantially higher for both SDD (7%) and OS (11%) without statistical significance between the groups (*p* = 0.06) [16]. In those studies where SDD adverse events can be attributed to the time interval from the end of the clinical observation period (6 h postprocedure) to 24 h postprocedure, these are listed in a separate column. Remarkably, 17 out of 19 prospective trials and 12 out of 12 retrospective studies/registries either did not report any major adverse events from 6–24 h or none at all, consistently highlighting the solid safety profile of SDD protocols. Consequently, the general safety precaution of one overnight stay would not have provided additional postprocedural safety in this selected patient group [17]. Those 2 studies reporting very low MACE rates of 0.26% [18] and 0.8% [19] within 24 h post-PCI (both due to stent thrombosis) are among the oldest in this review (2004 and 2005; Table 1 #2 and #4) and may not represent current PCI tools and P2Y12 inhibitor treatment.

### Meta-analysis

With the retrieved trial records we performed a meta-analysis representing current clinical practice focusing on radial access SDD. As shown in Fig. 1, we selected 9 clinical trials per standardized protocol from the initial 44 references. These trials (see Fig. 2) prospectively reported separate outcomes of 30-day MACE for both SDD and OS, and included data on radial access, the majority being performed exclusively with radial or ulnar access. Single-cohort studies and trials without specific data on access site were excluded. One study was based on the prospective Australian VCOR registry. The analysis itself, however, was done retrospectively [20], so the study was excluded. One of the RCTs was a substudy of the EXCEL trial focusing on left main interventions [21], therefore more high-risk procedures. In terms of frequency of MACE, there was no relevant difference to the other studies selected. Our meta-analysis contains a total of 23,017 patients, 2584 within RCTs and 20,433 within prospective 2‑cohort studies, and a total of 2405 SDD procedures. For the purpose of a brief analysis, only MACE data were extracted from the different sources.

The meta-analysis of all 9 prospective trials shows an odds ratio (OR) for MACE of 1.00 (95% CI 0.68–1.48; *p* = 0.21; I^2^ 8% Fig. 2a). The incidence of MACE was low in both investigated groups: 3% (49 of 1817) vs. 3% (87 of 3099; Fig. 2a). In one study no MACE occurred within 30 days, only a number of minor adverse events were detected [22], therefore, odds ratios were not estimable.

When extracting data only from the three RCTs, the meta-analysis showed an OR of 0.66 (95% CI 0.35–01.24; *p* = 0.19; I^2^ 0% Fig. 2b), indicating non-inferiority of SDD versus OS in carefully selected patients, which was consistent with our meta-analysis from all 9 prospective trials and the previously published meta-analysis data [9–12]. Again, the incidence of MACE was low in both investigated groups and numerically higher in the OS group: 2% (15 of 979) vs. 4% (58 of 1605), respectively.

## Data on safety of SDD in specific patients/settings

With growing experience of SDD PCPs and especially PCIs, some centers have extended the SDD concept to more challenging PCI indications, once again highlighting the robust safety profile of this treatment approach [23–28]:

### Complex PCI

Consistent with the findings of Small et al. ([27]; see 5.1), data on the feasibility of SDD following complex interventions taken from a recent Spanish multicenter registry have been published in 2019 [28]. A complex intervention was defined as either left main PCI, bifurcation with a 2‑stent technique, multivessel PCI with ≥ 2 vessels, rotational atherectomy, antegrade CTO or graft PCI. The SDD was feasible in 64% of patients with complex lesions versus 81% in patients with simple angioplasty [28]. The most common reasons for switching from SDD to OS were clinical symptoms (65%; chest discomfort, arrhythmias or at decision of physician in charge), suboptimal angiographic result (20%), vascular access complications (4%), crossover to femoral access (1.3%), rejection of SDD by patient or relatives (0.5%) and excessive contrast use (1%) [28]. In 791 patients out of 1047 discharged as intended, only a single MACE event occurred in the simple PCI group (*n* = 592), while no adverse events at all occurred in the complex PCI group (*n* = 199) at 24 h or 1 month [28]. A total of 20 of the 22 prospective studies in Table 1 and 19 of the 22 retrospective studies in Table 2 either specifically mentioned complex PCI procedures or PCI subgroups with features indicating more complex PCI (MV, bifurcation, LM, CTO, SVG), therefore showing an adequate representation of complex PCI procedures within the SDD cohorts studied.

### Older patients

Rao et al. analyzed data from 107,018 patients aged > 65 years from the CathPCI Registry in 2011. Among 1339 older patients discharged on the day of procedure, the rates of death or rehospitalization at 2 or 30 days postprocedure were not different from 105,679 patients admitted overnight [29]. Remarkably, consistent with the clinical standard at the time of publication, 96% of procedures in the SDD group and 98% in the OS group were performed with femoral access. Vascular closure devices were used in 65% and 50%, respectively [29].

An earlier retrospective cohort study examined the safety of SDD PCI performed via radial access between 1998 and 2001 in patients below and above the age of 75 years [30], including single vessel, multivessel and bypass graft interventions featuring lesions from type A to type C. In a self-reported outcome questionnaire detailing adverse events either from 0–24h or 1–30 days from discharge, there were no significant differences in major adverse events in 797 patients < 75 years and 146 patients > 75 years of age [30].

Older patients usually have more underlying chronic conditions, more advanced coronary and peripheral atherosclerosis, rendering radial/ulnar access more challenging. The recent expert consensus statements from the American College of Cardiology (ACC) [17] and the Society for Cardiovascular Angiography and Inteventions (SCAI) [31, 32] do not mention a strict age limit for SDD patients. In our experience, older patients aged > 75 years should be carefully screened considering their suitability for SDD treatment. Frequently not only adverse anatomical/vascular challenges are compromising SDD PCI, but also cognitive factors like the ability to comply with medication prescriptions and to organize reliable transport to and from the clinic on the day of the procedure.

### Rotational atherectomy

The feasibility of SDD rotational atherectomy (RA) has been considered critical by some authors [33]; however, in high-volume centers with experience in RA these interventions are increasingly being included in the array of SDD procedures. Of the prospective studies four ([28, 34–36]; Table 1) and two of the retrospective studies ([15, 37]; Table 2) in our review included small numbers of RA PCI procedures. This is consistent with a retrospective analysis of 4591 RA procedures from 2007 to 2014 in England and Wales published by Taxiarchi et al. [38]. The authors show an increase of SDD following rotablation from 6.7% in 2007 to 35.5% in 2014, representing 2.8% of the total uncomplicated elective PCIs in the registry. In terms of 30-day mortality, there was no superiority of OS compared to SDD [38]. Patients with MV PCI (14.0% SDD vs. 18.8% OS; *p* > 0.001) and LM PCI (8.0% SDD vs. 13.1% OS; *p* < 0.001) were found less frequently in the SDD cohort. On the other hand, SDD patients had PCI via radial access more frequently (48.3% SDD vs. 30.1% OS; *p* < 0.001), highlighting some of the selection criteria used by the majority of centers. The outcome with a 30-day MACE of 0.5% for SDD and 0.35% for OS (*p* = 0.409) showed an excellent safety profile with respect to this selection practice [38]. In the registry of SDD in complex lesions by Cordoba-Soriano et al., only 21 out of 1047 patients were treated with rotational atherectomy, and only 9 out of these (43%) were discharged on the day of the procedure [28]. The compatibility of highly aggressive PCI techniques with SDD programs should be critically evaluated by each interventional center depending on interventional experience and scope of procedures.

### Chronic total occlusions

The PCI of chronic total occlusions (CTO) generally involves a more aggressive setup with larger sheath diameter, high-tipload wires, microcatheters and dual access at least for the retrograde approach. Most CTO procedures also require multiple stents to cover longer segments of vessel occlusion. Therefore, the eligibility of CTO procedures for SDD has been viewed critically [33]. On the other hand, 10 of the 44 trials reviewed in Tables 1 and 2 included smaller numbers of CTO procedures, two studies [39, 40] (#17 and #20 in Table 2) specifically targeted CTO procedures on a SDD basis. A retrospective study from 2021 compared the 30-day outcome of 51 CTO interventions performed as SDD procedures with 122 performed with conventional OS. There were no demographic differences between both groups but non-SDD patients were more likely to have diabetes mellitus (non-SDD 51% vs. SDD 31%; *p* = 0.015) and arterial hypertension (non-SDD 89% vs. SDD 67%, *p* < 0.001), while SDD patients had a higher BMI and were more frequently smokers [40]. In SDD procedures, radial access was used as the single vascular access, while 17% of non-SDD patients had at least 1 femoral access. Outcome analysis showed an in-hospital MACE of 0% for SDD vs. 1.6% for non-SDD patients and a 30-day MACE of 0% and 1.6%, respectively. Antegrade wire escalation was the dominant crossing strategy for lesions in the SDD group; however, a multivariate logistic regression model showed that only diabetes mellitus and procedure time were independently associated with the decision to maintain a SDD strategy [40].

Similar results were found by Taxiarchi et al. in a retrospective longitudinal study from the UK, covering 7576 SDD cases versus 13,763 OS cases from 2007 to 2014 [39]. The percentage of SDD management among CTO procedures increased from 21.7% in 2007 to 44.7% in 2014. Patients in the OS cohort were more likely to have relevant medical histories, such as prior MI (OS 43.6% vs. SDD 39.2%; *p* < 0.001), prior coronary artery bypass graft (CABG) surgery (OS 16.3% vs. SDD 12.8%; *p* < 0.001), prior PCI (OS 35.6% vs SDD 31.9%; *p* < 0.001) and significant comorbidities as multivessel disease (OS 32.0% vs. SDD 29.7; *p* < 0.01) and arterial hypertension (OS 61.6% vs. SDD 59.4%; *p* < 0.01). Unadjusted 30-day mortality was lower for SDD patients (0.12% vs. 0.31% for OS; *p* < 0.01). After adjustment for clinical severity of cases, SDD was no longer independently associated with 30-day mortality (OR 0.54; 95% CI 0.25–1.15) [39]. Interestingly, while observed mortality rates for OS cases were well within expected mortality rates calculated from the British Cardiovascular Intervention Society (BCIS) risk score model [41], they were lower than expected for SDD patients throughout 7 of the 8 years of observation, although this trend did not reach the level of statistical significance [39]. The presence of ≥ 1 enabling strategies (dual access site, rotational atherectomy, intravascular ultrasound, and use of penetration catheters or microcatheters), was independently associated with overnight stay; however, the authors showed that in high-volume centers surgeons were more likely to treat more challenging cases than SDD [39].

Therefore, while the decision to include CTO interventions in the array of SDD procedures is consistent with the 2018 SCAI recommendations [31], it should certainly be critically appraised depending on the individual center’s expertise and caseload of CTO interventions.

### Left main coronary artery PCI

Left main coronary artery (LM) PCI was listed as an exclusion criterion for SDD by the 2009 SCAI position paper [33] and by the more recent 2020 SCAI position statement for PCI in ambulatory surgical centers [32] (as opposed to hospitals featuring in-house cardiac surgery departments). The 2021 ACC expert consensus document does not explicitly mention LM PCI as an exclusion criterion for SDD PCI [17]. Currently in Austria LM PCI is not routinely performed with SDD patient management; however, substantial experience does exist with SDD even in this high-risk setting. Out of 44 trials listed in Tables 1 and 2 14 included LM PCI procedures, 3 trials [21, 24, 42] specifically targeted LM PCIs in a SDD setting. A Canadian study compared the outcome of elective LM PCI in 267 patients treated as SDD to 194 patients with OS [42]. Patients in the SDD group were younger (70.9 ± 10.1 years versus 73.4 ± 10.8 years), more frequently had prior cardiac catheterization and showed a larger percentage of protected LM stenoses (51.7% versus 35.1%). The composite primary endpoint of 30-day mortality, myocardial infarction and rehospitalization was significantly lower in the SDD group (OR 4.3; 95% CI 1.1–6.0, *p* = 0.002) [42]. This remarkable outcome depends, at least in part, on the inherent selection bias of SDD suitable patients following current recommendations. Conversely, however, it does underline the robustness of the SDD concept with correct patient selection even in complex interventions. One of the most remarkable findings regarding the standard OS as a safety precaution was, once again, the timing of complications: All MACEs occurred beyond 48 h postprocedure, therefore OS would have provided no additional safety benefit [42].

A large retrospective multicenter analysis evaluated the outcome of 6452 LM PCI interventions in England and Wales from 2007 to 2014 [24]. The authors found that SDD treatment after LM PCI had almost doubled during the study period (all LM PCI 19.9–39.8%; unprotected LM 20.7–41.4%) in parallel with an increase in procedural complexity including rotational atherectomy and multistent strategies. Nevertheless, SDD was not associated with an increase in 30-day mortality in general LM PCI procedures (OR 0.70, 95% CI 0.30–1.65) and in unprotected LM PCI (OR 0.48, 95% CI 0.17–1.41) [24]. Consistently, a subgroup of 100 LM interventions within the prospectively randomized EXCEL trial were performed on a SDD basis ([21]; see Table 1 #21). Compared to 835 OS LM procedures, there were no significant differences in MACE at 30 days (4.0% SDD vs. 5.0% OS, adjusted OR 0.52, 95% CI 0.12–2.22; *p* = 0.38) or 5 years (20.6% SDD vs. 22.1% OS, adjusted OR 0.72, 95% CI 0.40–1.29; *p* = 0.27).

In summary, LM PCI in carefully selected clinically stable patients without excessive calcifications appears to be feasible and safe in a SDD setting; however, it must be taken into account that the multicenter study cited above reported substantial heterogeneity of LM PCI frequencies among different clinics [24] and that the favorable outcome is largely driven by high-volume centers with great expertise in complex and LM PCI. The individual decision to offer SDD for a LM PCI patient should be made individually involving the interventional team’s level of expertise, the operator’s preference and the clinical course during and after the intervention.

## Patient satisfaction

Patient preference should be taken into account when scheduling invasive procedures. There is a well-documented patient preference towards radial access for PCP, which leads to less discomfort, less frequent hematomas and better quality of life post procedure [8, 43].

Across different countries, different study settings and decades of coronary interventional experience SDD after PCP has been shown to be the preferred treatment mode as opposed to OS [20, 37, 44–49], satisfying another clear patient preference for short hospital stay, earlier and easier ambulation.

In a randomized controlled trial on quality outcomes from 2013, 79% of patients randomized to SDD post-PCI were satisfied with their discharge timing compared to 49% randomized to next day discharge (*p* < 0.01) [46]. At 30 days only 9% of SDD patients reported that they would have preferred a longer hospital stay, whereas 37% of the OS group would have preferred an earlier discharge. Clopidogrel adherence and rate of clopidogrel discontinuation were similar in both groups (SDD 12% vs. OS 13%) at 30 days post-PCI [46]. Similar findings were reported in a study from 2021 including in-depth interviews with patients and family members [48]. The SDD was preferred by the majority of patients and family members, the absence of lengthy surveillance on the ward was perceived as very positive, some patients even felt their heart condition to be less concerning due to the fact that they were free to return home post procedure. In most cases of negative experiences, proper instructions for patients and families were missing, either considering the details and time of discharge or prescribed home medication [48].

## Discussion

We present a structured review of 44 SDD PCP studies and a comprehensive meta-analysis of 9 prospective trials focusing on radial access. As indicated above, it should be noted that 20 out of 22 prospective trials (Table 1) and 19 out of 22 retrospective studies (Table 2) reported specific features of complex PCI including MV, bifurcation, lesion type C, LM, CTO and RA, therefore providing a realistic representation of the everyday catheterization laboratory case complexity.

Confirming data from previous reviews our assessment of a large number of more recent SDD studies with radial access shows a favorable safety profile with event rates mostly showing lower single digit percentages. Compared to data from the CathPCI registry, a part of the American National Cardiovascular Disease Registry (NCDR) (2016 Q4–2017 Q3, cited in [33]), including results from > 600,000 patients without ST-segment elevation MI or bypass surgery, where the overall incidence of in-hospital complications was 4.8% (stroke 0.2%, bleeding within 72 h 1.4%, pericardial tamponade 0.9%, acute kidney injury 0.2%), the safety profile for SDD PCI is quite favorable. Considering the timing of complications and related safety concerns our review confirms the appropriate length of follow-up 6 h postradial access PCI. Small et al. retrospectively analyzed the complication rate and timing of radial access PCI procedures in 1174 patients with clinical or procedural features rendering them intermediate or high-risk patients [27]. After treating 1543 lesions, 90% of which were type B2 or C, bleeding complications occurred in 13 patients (1.2%) within 6 h (12 of 13 being minor) and a total of 8 patients (0.7%) suffered transient neurological symptoms. A further 6 patients (0.5%) had to be transferred to urgent bypass surgery due to intraprocedural complications. Apart from the remarkably low complication rate in a higher risk patient collective, once again the absence of adverse events between 6 and 24 h was confirmed [27]. In their trend analysis of 819,091 procedures from 2009 to 2017, Bradley et al. consistently showed the absence of any association between discharge policy and 30-day mortality, which was 0.2% for both OS and SDD [4].

It must be acknowledged that in numerous studies exclusion or conversion rates from SDD to OS were particularly high, extreme outliers showing 90% (Table 1 #14; [50]) and 70% (Table 2 #18; [40]). This was caused rather by strict criteria within the trial or operator concern than by actual complications. Reiterating clinical experience from outpatient clinics in Austria, conversion rates from SDD to OS are around 5% (single center experience), mostly due to detection of three vessel CAD with indication for urgent bypass surgery or out of an abundance of caution in the absence of clinical symptoms.

Our meta-analysis of nine prospective trials showed a low incidence rate of MACE in SDD versus OS PCI (3% vs. 3%; OR 1.00), with no additive risk connected with outpatient management. This is consistent with four previous large meta-analyses deriving outcome data for SDD PCI [9–12]. As discussed above, it should be noted that due to their time of publication some of the older meta-analyses incorporated trials still featuring 100% femoral access and routine application of GPIIb/IIIa antagonists during PCI.

A large meta-analysis of 12,803 patients from 37 studies, including 7 randomized trials (radial access 60.8%) and 30 observational studies (femoral access 70.0%) found no significant differences in their co-primary endpoints (see Table 3; [10]). Patients randomized into the SDD group within the RCTs listed could actually be discharged on the day of procedure in 87.3% of cases, the most common reasons for discharge deferral being access site complications (33%), physician preference (30%), patient preference (17%) and recurrent chest pain (11%). In observational studies 71.7% of the cumulative 14,032 patients eligible for SDD were discharged on the same day [10]. Out of 15 deaths reported in 30 observational studies summarized by Brayton et al. all cases with a documented time of the event occurred beyond 24 h postprocedure, at a time when both SDD and OS patients would have left the hospital [10].

**Table 3:** Evidence from meta-analyses

Study	Reference	Number of RCT studies	Number of OBS studies	Sample size SDD	Sample size OS	Percentage radial access	Percentage femoral access	Endpoints	Outcome
Brayton et al. 2013	[10]	7	–	1256	1482	60.8%	39.2%	Death, MI, TVR, stroke, vascular and bleeding complications	87.3% successful SDD per protocol
									No differences for composite primary endpoints: (prim. EP: 7.17% SDD vs. 6.07% OS; OR 0.90 (95% CI 0.43–1.87; *p* = 0.78); major bleeding/vascular complications: 1.88% SDD vs. 1.29% OS; OR 1.69%; 95% CI 0.84–3.40; *p* = 0.15)
		–	30	10,065	3967	30%	70%	Death, MI, TLR, major bleeding, vasc complications	71.2% successful SDD per protocol
									Primary endpoint at 1.00%, bleeding complications 0.68%. Documented timing of fatalities > 24h postprocedure
Abdelaal et al. 2013	[9]	5	–	1023	1016	49.2%	50.8%	Death, MI, MACE, rehospitalizations	80–88% successful SDD per protocol
									Complications 6.5% (SDD) vs. 5.5% (OS)
		–	8	3156	106,635	2.6%	97.4%	Death, MI, MACE, rehospitalizations	Complications 4.7% (SDD) vs. 9.6% (OS)
Bundhun et al. 2017	[11]	8	–	1598	1483	ND	ND	Death, MI, MACE, bleeding complications	SDD vs OS mortality: OR 0.22 (95% CI 0.04–1.35, *p* = 0.10); MI: OR 0.68 (95% CI 0.33–1.41; *p* = 0.30); MACE: OR 0.45 (95% CI 0.20–1.02, *p* = 0.06). No significant differences SDD vs OS for major endpoints
Lu et al., 2019	[12]	3	–	575	467	31%	69%	MACE (death, MI, stroke, repeat revascularization), arrhythmia, major/minor bleeding, hematoma, rehospitalization	MACE (OR: 0.75, 95% CI: 0.31–1.79; *P* = 0.51), mortality (OR: 0.26, 95% CI: 0.06–1.06; *P* = 0.06), stroke (OR: 1.46, 95% CI: 0.72–2.94; *P* =0.29), arrhythmia (OR: 1.30, 95% CI: 0.64–2.63; *P* =0.47), hematoma (OR: 1.00, 95% CI: 0.60–1.66; *P* = 1.00), major bleeding from access site (OR: 1.68, 95% CI: 0.22–12.85; *P* =0.62) no significant differences
		–	8	21,112	140,999	4%	96%*		

Current data on PCP SDD safety from meta-analyses

*CI* confidence interval, *EP* statistical endpoint, *MACE* major adverse cardiovascular events, *MI* myocardial infarction, *ND* no data available *OBS* observational studies, *OR* odds ratio, *OS* overnight stay, *RCT* randomized controlled trials, *SDD* same-day discharge, *TLR* target lesion revascularization,

* 95% of transfemoral cases within observational trials attributable to a single study Rao et al. [29]

A second systematic review confirmed these results based on pooled data from 5 RCTs and 8 observational studies published from 1999 to 2011 with a total of 111,830 patients to compare the outcome of SDD vs. OS procedures [9]. In the 5 RCTs complications, defined as total complications, major adverse cardiovascular events and rehospitalization within 30 days of PCI, occurred in 6.5% in SDD procedures vs. 5.5% in the OS group (OR 1.20, 95% CI 0.82–1.74). In the compilation of observational studies complications were reported in 4.7% of the SDD procedures versus 9.6% in the OS group (OR: 0.67, 95% CI 0.27–1.66) [9]. Radial access was used in 46.2% of procedures in randomized trials and in only 2.6% of procedures in observational studies, mostly caused by a single study with 107,018 patients, performed with femoral access in 97.65% of all cases, which counterbalanced all other studies within the pool and caused significant statistical heterogeneity. Although trials on SDD PCI involved some level of patient selection, the entire pooled population in the systematic review was male in 64%, diabetic in 32%, had treated hypertension in 79%, prior PCI in 39% and status post-bypass surgery in 23%, therefore comparable to the classical patient profile in interventional cardiology [9].

It must be acknowledged for our data compilation, as for previous large reviews or meta-analyses of multiple trials [9, 10], that the resulting SDD patient collective and array of procedures are rather heterogeneous. Remarkably, MACE event rates across this spectrum remained very low, even in trials exclusively enrolling CTO [39] or LM [21] PCI procedures. This may serve as proof for the efficacy of the patient selection process for SDD procedures, excluding significant comorbidities, such as left ventricular ejection fraction (LVEF) < 30% or chronic kidney disease (CKD) which have been shown to be significant predictors of periprocedural complications [10, 51, 52]. On the other hand, the multitude of SDD studies evaluated in reviews and meta-analyses have utilized different sets of patient selection criteria and one of the major goals identified by many reviewers was establishing universal evidence-based patient selection recommendations [9, 10]. After the SCAI [32, 33] and the ACC [17] published their own recommendations, this updated review and meta-analysis serves as a foundation for the Austrian Society of Cardiology’s practice recommendations for SDD PCPs [3].

## Abbreviations

ACS: Acute coronary syndrome
AKI: Acute kidney injury
CAD: Coronary artery disease
CIN: Contrast-induced nephropathy
CTO: Chronic total occlusion
DA: Diagnostic angiography
DAPT: Dual antiplatelet therapy
GFR: Glomerular filtration rate
LM: Left main coronary artery
MACCE: Major adverse cardiovascular or cerebral event
MACE: Major adverse cardiovascular event
MV: Multivessel
ND: No data
n. s.: Not statistically significant (*p* < 0.05)
NS: Not specified
NSTEMI: Non-ST-segment elevation myocardial infarction
OBS: Observational study
OCT: Optical coherence tomography
OR: Odds ratio
OS: Overnight stay
PCI: Percutaneous coronary intervention
PCP: Percutaneous coronary procedures
RA: Rotational atherectomy
RCT: Randomized controlled trial
SDD: Same-day discharge
STEMI: ST-segment elevation myocardial infarction
SVG: Saphenous vein graft
TLR: Target lesion revascularization
TTE: Transthoracic echocardiography
TVR: Target vessel revascularization
